# Carbon monoxide in intensive care medicine—time to start the therapeutic application?!

**DOI:** 10.1186/s40635-020-0292-8

**Published:** 2020-01-09

**Authors:** Ulrich Goebel, Jakob Wollborn

**Affiliations:** 1 Department of Anaesthesiology and Critical Care, St. Franziskus-Hospital, Hohenzollernring 70, 48145 Münster, Germany; 2 Department of Anaesthesiology and Critical Care, Medical Centre - University of Freiburg, Faculty of Medicine, Freiburg im Breisgau, Germany

**Keywords:** Carbon monoxide, Haeme-oxygenase-1, Acute respiratory distress syndrome, Idiopathic pulmonary fibrosis, Acute kidney injury, Extracorporeal circulation, Transplantation

## Abstract

Carbon monoxide (CO) is not only known as a toxic gas due to its characteristics as an odorless molecule and its rapid binding to haem-containing molecules, thus inhibiting the respiratory chain in cells resulting in hypoxia. For decades, scientists established evidence about its endogenously production in the breakdown of haem via haem-oxygenase (HO-1) and its physiological effects. Among these, the modulation of various systems inside the body are well described (e.g., anti-inflammatory, anti-oxidative, anti-apoptotic, and anti-proliferative). Carbon monoxide is able to modulate several extra- and intra-cellular signaling molecules leading to differentiated response according to the specific stimulus. With our growing understanding in the way CO exerts its effects, especially in the mitochondria and its intracellular pathways, it is tempting to speculate about a clinical application of this substance. Since HO-1 is not easy to induce, research focused on the application of the gaseous molecule CO by itself or the implementation of carbon monoxide releasing molecules (CO-RM) to deliver the molecule at a time- and dose dependently safe way to any target organ. After years of research in cellular systems and animal models, summing up data about safety issues as well as possible target to treat in various diseases, the first feasibility trials in humans were established. Up-to-date, safety issues have been cleared for low-dose carbon monoxide inhalation (up to 500 ppm), while there is no clinical data regarding the injection or intake of any kind of CO-RM so far. Current models of human research include sepsis, acute lung injury, and acute respiratory distress syndrome as well as acute kidney injury. Carbon monoxide is a most promising candidate in terms of a therapeutic agent to improve outbalanced organ conditions. In this paper, we summarized the current understanding of carbon monoxide’s biology and its possible organ targets to treating the critically ill patients in tomorrow’s ICU.

## Background

Being an odorless and difficult to sense gas, carbon monoxide (CO) was usually referred to as the “silent killer” with a myriad of published and unpublished fatal accidents, mostly due to incomplete combustion of organic material or explosions [1]. The high affinity of CO to hemoglobin was used as one possible explanation for the toxic effects [2, 3]. Although different symptoms of CO intoxications were seen (ranging from headache and fatigue to nausea and vomiting, confusion, and convulsion and finally death), it took more than 50 years to prove Paracelsus’ maxim to be true: “only the dose makes the poison.”

Carbon monoxide was recognized and first described in 1925 to be more than just a toxic, odorless and thus very dangerous gaseous molecule [4–7]. Since its discovery as an endogenously generated product in the degradation process of haem, a multitude of in-vitro and in-vivo experiments have been performed to analyse its effects in a variety of systems and diseases and shed light on the impact as well as the molecular mechanism of this interesting gas [8–16]. The finding, that the catalytic degradation and conversion of hemoglobin into its parts (i.e., biliverdin, iron and carbon monoxide) is an enzyme-triggered process directed research into a new direction. Tenhunen and Schmidt first identified the enzyme responsible to produce CO endogenously: hemoxygenase (HO) [17]. Haem-oxygenase-1 and -2 (HO-1 and -2) have been demonstrated to be the (stress-) inducible and constitutive isoforms of the rate-limiting enzyme, responsible to produce CO [18]. While the knowledge of significance only emerged slowly over the years, it was in 1999 that the case of a child with proven HO-1 deficiency was reported, suffering from a variety of organ dysfunction [19]. Since CO is thought to be the crucial product of the HO breakdown, a generation of scientist was in search for non-toxic but yet potent HO-1 inducible drugs. Among various agents in question, anesthetics (e.g., isoflurane and sevoflurane) were found to be capable of a significant HO-1 induction providing not only an upregulation of HO-1, but also organ protection, while being clinically safe [20–22].

**Fig. 1:**
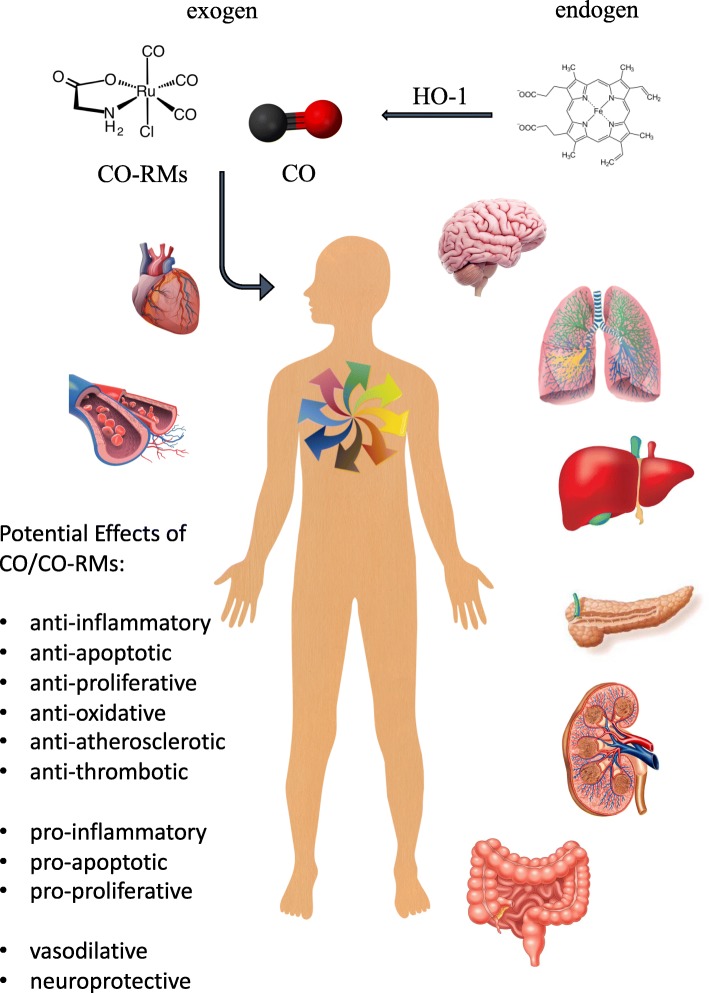
Carbon monoxide may be administered to the human body in three different ways: First by the induction of haem-oxygenase-1 to a relevant level, second by the inhalation of carbon monoxide itself, and third by the intravenous injection or oral intake of any kind of carbon monoxide releasing molecule. Possible target organs include (but are not limited to) the brain, the heart, the lungs, the liver, the kidney, the pancreas, the gut, and the vessels. The effects of carbon monoxide application may be (single or more than just one): anti-inflammatory, anti-apoptotic, anti-proliferative, anti-oxidative, anti-atherosclerotic, anti-thrombotic, vasodilative, neuroprotective, as well as pro-inflammatory or pro-apopototic

The pure substance of carbon monoxide itself may alter various diseases in all kinds of experimental physiological systems, settings, and target-organs (i.e., anti-inflammatory, anti-apoptotic, anti-oxidative, anti-proliferative, and vasodilative etc.); see Fig. 1 [14, 23–26]. These include potential disease, which may be of interest in the ICU (pulmonary arterial hypertension [PAH], acute respiratory distress syndrome [ARDS], acute kidney injury [AKI], sepsis, transplant settings, and the use of extracorporeal circulation units [ECMO, ECLS]) [27]. However, the straight clinical use of CO via inhalational administration—which would be the logical consequence of the above said—is currently difficult to implement. Due to the relatively low solubility of molecular CO in water (about 1 mM), its distribution and allocation to target tissues seems limited. In order to reach sufficient concentration at target side, enormous concentrations of inhaled CO would be needed. Apart from this, CO reacts relatively fast with other serum proteins, which in turn limits its potential (inter-)action at the target organ side (as long as it is not the pulmonary system).

The direct high toxicity due to the inhalation of even putative “low doses” of carbon monoxide triggered new methods to apply defined doses at a pre-set organ or tissue within a defined time-window [28]. In recent years (and beside the other well established gaso-transmitters like nitric oxide, hydrogen sulfide or argon), carbon monoxide has attracted rising attention, since scientists were able to design and produce specific small molecules, so called carbon monoxide releasing molecules (CO-RMs), meeting the specific requirements such as solubility, low toxicity, and triggered release of CO [15, 29–33]. CO-RMs show comparable effects (e.g., anti-inflammatory impact) as the carbon monoxide gas [34]. The well-known preference of CO toward metal compounds (e.g., the iron molecule in hemoglobin) and the knowledge about transition metals in organic chemistry predicted the way of the development of CO-RMs. Although the first CO-RMs were able to deliver CO at controlled amounts and toward specific tissue, the problem of the toxicity of the transition metal backbone (e.g., ruthenium in CORM-2 or molybdenum in ALF186 and ALF062) are still a problem. In contrast, CORM-A1 presented with a boron containing molecule and a longer half-life of approx. 21 minutes [35].

Recently, a new class of enzyme-triggered CO-RMs (ET-CORM) has been introduced. Enzyme-triggered CO-RMs have been designed in order to improve target-tissue concentration of CO since ET prodrugs are activated time-dependently by serum esterases achieving site-specific CO-release [30]. Moreover, the modification of the outer ligand sphere by peptides may support the controlled release of CO at the target side [36, 37]. While these substances have not been analyzed in-vivo up-to-date, there is some evidence about their potential in HUVECs, displaying the activation of Nrf-2 and HO-1 by counteracting TNF-α induced inflammation and inhibiting VCAM-1 [38, 39].

Although all kinds of CO-RMs may deliver CO in a multitude of different ways and kinetics due to different stimuli [28, 40, 41], the beneficial effects of CO on organ function still need to be evaluated in the context of the whole organism. Very special attention is necessary in terms of safety issues regarding the metabolic fate of either the donor molecule, the specific transition metal backbone, and CO itself [42, 43].

This review will discuss the potential human target organs, mechanisms, and strategies to implement carbon monoxide independent of the source of administration (the gaseous molecule CO or any kind of CO-RM) according to the published literature of CO-administration in humans to the benefit of disease prevention and therapeutic application at the level of a human intensive care medicine setting.

### Carbon monoxide biology and mechanism of action

Both carbon monoxide and it’s artificial releasing molecules exert a wide range of biological effects including a variety of different intracellular pathways. Important to know that the transition metals in CO-RMs may affect the above outlined beneficial effects of CO. The main effects of CO may be summarized as follows: anti-inflammatory [8], anti-thrombotic [44], anti-oxidative [45], anti-proliferative [46], anti-apoptotic [11], anti-atherosclerotic [47], neuroprotective [11, 48], and vasodilative [49]. Of note, CO may dose-dependently exert certain “pro”-effects: pro-inflammatory, pro-apoptotic, and pro-proliferative, depending on the individual setting [50–52].

**Fig. 2:**
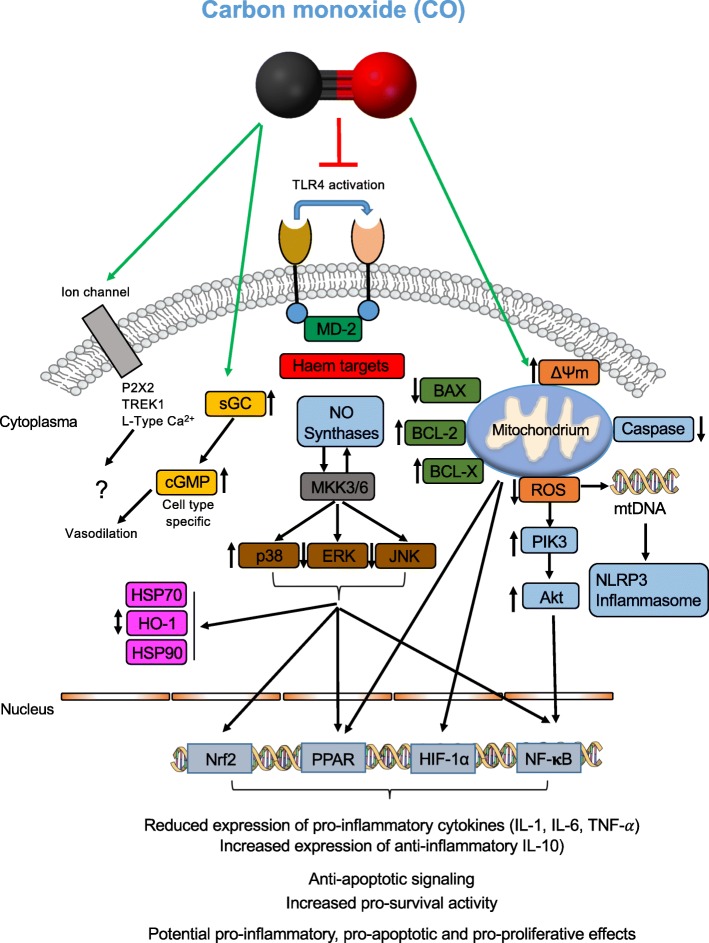
Schematic of some known molecular targets, channels, receptors, and intracellular protein structures, that may be altered in the context of carbon monoxide or CO-RM application. The anti-inflammatory, anti-apoptotic and pro-survival signalling may be of interest in treating critical illnesses in the ICU

The molecular mechanism is well understood—a summary is given in Fig. 2. Of note, not all known target proteins or structures have been implemented in this figure to facilitate the understanding of the main processes. Carbon monoxide preferably targets haem-containing proteins, such as hemoglobin, the mitochondria oxidases, the soluble guanylyl cyclase, or the enzymes necessary for reactive oxygen species generation [15, 53]. The anti-inflammatory effects have been well described by Otterbein et al. He was able to demonstrate that CO differentially affects the cytokine system, decreasing the expression of pro- and increasing the expression of anti-inflammatory interleukins [14]. These findings are consistent throughout the literature in different models, including LPS-stimulated macrophages microglia cells [54, 55]. Carbon monoxide confers these anti-inflammatory effects through the activation of the mitogen activated protein kinases (p38, ERK, and JNK) in response to different types of stressors [16]. In detail, CO regulates its anti-inflammatory and its anti-apoptotic effects via an activation of the MKK3/p38β MAPK pathway [12, 14]. The soluble guanylyl cyclase (sGC) may be affected by CO and contributes to the alteration of nitric oxide synthases (NOS), which in term modifies the expression of the MAPK [56, 57]. The regulation of JNK or ERK in the context of carbon monoxide application has been described to varying extents in different cell types and conditions [10, 26]. Part of the anti-inflammatory modulation may be the inhibition of TLR-4 trafficking while interacting with caveolin-1 at the outer plasma membrane [58, 59]. The exact intracellular mechanisms of carbon monoxide will not be discussed in detail; the specific literature will give answers [16].

## Potential targets of CO in intensive care medicine

Almost every organ system and tissue has been subjected to CO treatment in the last decades [40]. All species have been part of a research that well evaluated the effects of any disease ranging from malaria to stroke and from heart disease to compartment syndromes and breaking down the exact molecular mechanism of its terms of action [60–63]. Several of these studies point toward important conditions that are most commonly treated on intensive care units. While there were many promising studies in human beings, most of them failed to prove effectiveness, did not reach the statistical significance, or have been withdrawn for a variety of reasons [64–74] (see Table 1). Apart from that, all studies were able to provide data, that the administration of low-doses of carbon monoxide seems safe. Carbon monoxide concentration, duration of application, and hemoglobin levels during or after inhalation did not exceed FDA approved limits.

**Table 1:** History of carbon monoxide inhalational clinical trials including healthy volunteers and patients. The lower part represents the studies, which have been withdrawn for various reasons. (This list is not exhaustive)

Ref.	Year	First author	Number of participants	Carbon monoxide dose	CO-Hb	Outcome/marker
52	1973	R.D. Steward	Not reported	100 ppm for 8 h	11–13%	Feasibility study
53	1975	J.E. Peterson	22 volunteers	50, 100, and 200 ppm for 5.25 h	1–20%	Feasibility study, check of Coburn-Forster-Kane equation
55	1997	M. Hausberg	10 volunteers	30 min of 1000 ppm followed by 30 min of 100 ppm	8.3 ± 0.5%	Acute sympathetic and hemodynamic effects, no differences between groups
54	2001	Ren	11 subjects	4000 ppm until CO-Hb = 10%	9.7 ± 0.1	Respiratory response to prolonged hypoxia, no differences between groups
56	2001	Zevin	12 healthy smokers	1200–1500 ppm and smoking of at least 20 cigarettes per day for 7 days	5 ± 1%	No differences in blood pressure or respiratory rate; analysis of platelet factor 4 and urine epinephrine and norepinephrine
58	2005	Mayr	9 volunteers	500 ppm for 1 h after intravenous LPS injection (2 ng/kg BW)	6.5–7.7%	TNF, IL-1, IL-6, IL-8 concentrations in plasma showed no differences
62	2005	Resch	15 volunteers	500 ppm for 1 hour	8.5 ± 0.9%	Significant increase in retinal and choroidal blood flow
59	2007	Bathoorn	20 COPD patients	100–125 ppm CO for 2 h on 4 consecutive days	4.5%	Trend in the reduction of sputum eosinophils, improvement in response to metacholine
57	2009	Rhodes	6 subjects	100 ppm for 1 h for 5 days	3.3 ± 0.6%	Significant increase in SOD, HO-1, mitofusin, ATPase-6 and COX-1 proteins due to CO inhalation
60	2015	Pecorella	19 subjects	200 ppm for 1 h for 5 days	5.4 ± 0.8%	Significant increase in muscle citrate synthase, mitochondrial density and GLUT4
61	2016	Ryan	18 volunteers	1.2 mL/kg BW for 30 s for 10 days	4.4 ± 0.4%	No changes in haemoglobin mass, aerobic performance or peak power exercise tolerance
				Studies withdrawn		
	2007	NCT00531856	%	5.97 mg/L of carbon monoxide in 30% oxygen post transplantation (3 different doses)	n.a.	Primary outcome: Evaluate the safety and tolerability, secondary: incidence of delayed graft dysfunction (study withdrawn)
	2012	NCT01523548	%	150 ppm for 3 h for 3 weeks	n.a.	Primary outcome: Evidence of a decrease in pulmonary vascular resistance post-therapy (Study withdrawn)
	2010	NCT01050712	%	Depending on a healthy control group	n.a.	Primary outcome: incidence and duration of postoperative ileus (Study withdrawn)
	2012	NCT01727167	%	200 ppm for 1 h	n.a.	Primary outcome: Biochemical Markers for Mitochondrial Biogenesis in aortic valve surgery (Study withdrawn)

### Carbon monoxide in pulmonary diseases

Pulmonary arterial hypertension (PAH) is a rare disorder which is mainly characterized by a slow but ongoing chronic inflammation and thus obliteration of small pulmonary arteries. This leads finally to an increase in pulmonary arterial pressure and resistance and consequently to the failure of the right ventricle. The understanding of the underlying mechanism evolved well over recent years—nevertheless, therapies including receptor antagonists against endothelin or PDE-5 inhibitors only show moderate effectiveness, especially in terms of protecting the right ventricle [75]. Since no cure exists to treat PAH causal, prognosis remains very poor unless lung transplantation is an option. Concerning the anti-inflammatory effects of carbon monoxide, an option may be the inhalation of CO regarding its direct impact in the pulmonary system.

In 2012, a clinical trial was initiated (NCT01523548: carbon monoxide therapy for severe pulmonary arterial hypertension) which unfortunately was withdrawn sometime later. Drug administration of CO was considered to be in an increasing manner, starting with 150 ppm × 3 h once weekly (week 1), 150 ppm × 3 h twice weekly (week 2), and ending with 150 ppm × 3 h three times a week (week 3–16). The primary outcome was clearly defined as the evidence of a 20% decrease in pulmonary vascular resistance post-therapy when compared to pre-therapy value. While not being executed, this study plan represents one of the promising options to treat a rare but severe disease, especially in patients where left heart failure is not the reason for PAH. The anti-inflammatory effects of CO would most likely directly interact with the endothelial cells to counteract ongoing obliteration.

### Acute respiratory distress syndrome

The acute respiratory distress syndrome (ARDS) is a common complication of a variety of diseases requiring intensive care treatment. It may be characterized by a severe acute lung injury (ALI) and hypoxemic respiratory failure. The overall mortality rate ranges up to 40% [76]. Among other risk factors, sepsis is considered a major contributor for ARDS development during ICU stay [77]. Since other treatment options failed to provide sufficient data to reduce morbidity and mortality, there is a strong need to develop new strategies in treating both ALI and ARDS.

Experimental data showed promising results regarding the application of low-dose carbon monoxide in preclinical models of ARDS [16, 59, 78–82], including some mechanistic considerations, such as the induction of autophagy, the inhibition of mitochondrial dysfunction, inflammasome activation, and an accelerated resolution of the inflammation via special pro-resolving mediators [80, 83–85].

Recently, Fredenburgh and colleagues extended their previous research in baboons and published the first-in-man study regarding the effects of inhaled CO in sepsis-induced ARDS patients (NCT 02425579) [86]. In this placebo-controlled clinical trial, 12 patients were enrolled. They received treatment with 100 or 200 ppm inhaled CO respectively, aiming to achieve an algorithm-specified CO-Hb of 6–8%. The authors were able to show that CO inhalation is safe in their setting and the CO-Hb may not only be measured but can be predicted accurately using the Coburn-Forster-Kane equation. Furthermore, the author found a significant reduction in mtDNA, while there was no reduction in any other biomarker (IL-18 or RIPK3). Although the cohort was small in this phase-1 trial, there were no statistical differences in PaO_2_/FiO_2_ ratio, the oxygenation index, lung injury score, lactate, or the SOFA score. These results are most promising and show not only the feasibility and safety of inhaled CO, but the translation of pre-clinical data into a relevant clinical setting. For sure, further studies are needed to confirm these results in a larger population.

### Idiopathic pulmonary fibrosis

Idiopathic pulmonary fibrosis (IPF) is a progressive interstitial lung disease, mainly found in older adults and limited to the lungs. Fibroblast proliferation irreversibly destroys the cellular architecture of the lungs, finally leading to respiratory failure and death [87]. While different treatment options and therapeutic agents are standard treatment on intensive care units [88], there has been only little progress over the last years, with the transplantation of the lungs as the only cure in these patients. Apart from environmental and epigenetic factors, the underlying molecular pathway of the disease is quite well understood, suggesting an imbalance between pro- and anti-fibrotic mediators. Part of this imbalance may be exerted by inflammatory proteins such as TNF or IL-1 [87].

Carbon monoxide was able to suppress bleomycin-induced pulmonary fibrosis in as murine model, modulating the mitogen-activated protein kinases as the commonly known targets [89]. Recently, Rosas and colleagues published their results of a phase-2 clinical trials (NIH U01HL105371) in 58 patients with IPF. The aim was to show that low doses of CO (100 and 200 ppm) inhaled twice a day for 12 weeks would decrease IPF-specific gene expressions (such as MMP7 levels) in the peripheral blood as a sign of decreased disease progression [90]. Unfortunately, there were no differences in the treatment arms regarding the clinical effects of carbon monoxide.

### Carbon monoxide in acute kidney injury

Acute kidney failure (AKI) presents a critical illness with increased morbidity and mortality which is common on every ICU these days, especially in patients presenting with progressive comorbidities and rising age. While a variety of classifications (RIFLE, AKIN, KDIGO) are known, creatinine levels are the most effective contributor to this graduation, showing the extent of excretory defects [91]. Renal replacement therapy should be avoided as long as possible and treatment options should include experimental strategies such as carbon monoxide to treat one cause of AKI—e.g., renal ischemia and reperfusion injuries. Data exists that renal IRI may be effectively counteracted by low dose administration of carbon monoxide [92].

The authors were able to show that cytochrome C was suppressed during ischemia while being at normal levels due to CO inhalation. This may be of special interest in transplant settings, where ex-vivo cold storage is still common. Hanto and colleagues developed a pig kidney allograft model [93]. They demonstrated that the administration of CO reduced tubular necrosis and apoptosis. On a molecular basis, they analyzed significant changes in the expression of p-selectin and an increase in repair mechanisms. An additional microarray confirmed data of studies in other species, showing that CO was able to reduce the expression of MCP-1. While apoptotic markers were decreased in their experiments, the authors conclude that low dose CO may be an alternative treatment option for delayed graft function after transplantation.

Yoshida supplemented kidney transplantation in a porcine setting with CO, reaching CO-Hb levels of 7 ± 1.5%. Survival rates in the CO-treated group reached 100%, while only 80% survived in the control group. Analysis of the graft 14 days after transplantation revealed a significantly improved histological score, including less tubular damages, fewer focal fibrotic changes, and lower number of infiltrates in the kidneys. The expression of TGF-beta and p-Smad3 was reduced due to CO application. The authors therefore concluded that the ex-vivo exposure to CO during cold storage may be an easy alterative and an absolutely safe strategy to reduce IRI in renal transplantation [94].

Neto and coworkers analyzed the classical targets of CO (i.e., mRNA for IL-6, IL-1beta, TNF-alpha, ICAM-1, heme oxygenase-1, and inducible nitric oxide synthase) in a model of kidney transplantation in rats. Exposure of 250 ppm CO for 1 h before and 24 h after transplantation revealed lower expression of mRNA in these proteins. Moreover, the survival rate was significantly higher in CO treated rats than in the control group (60 vs. 25 days). Histopathology revealed the same results as in the other experimental protocols: lower injury scores regarding acute tubular necrosis, interstitial hemorrhage, and edema [95]. These findings are in line with the data of Goebel, who demonstrated reduced renal injury after cardiopulmonary bypass and subsequent IRI of the kidney s[9].

Concerning the clinical application of carbon monoxide in humans, there was a most promising clinical trial, which started in 2007 (NCT00531856; Safety and Tolerability Study of inhaled carbon monoxide in Kidney Transplant Patients). In contrast to the other studies, which aimed to inhale CO in a fixed dose (e.g., 250 ppm), the investigators used the COVOX device, a small tool giving a specific dose of CO dependent on the bodyweight and the breathing cycle to the participant. Unfortunately, the study was withdrawn early. The primary outcome was to clear safety issues in terms of the new application method.

### Carbon monoxide in sepsis

Sepsis is among the leading causes of death and in-hospital mortality throughout the world [96]. While the cause of sepsis may be different (e.g., pneumogenic sepsis, urogenital sepsis, etc.), the best strategy to treat the individual septic cause (intravenous fluid load versus vasopressor treatment) remains uncertain. As our society turns to older ages, septic conditions are more likely to occur. Lately, artificial intelligence was introduced to help physicians to find the best treatment option [97]. The pathophysiology of sepsis is very complex, including interactions of infecting microorganism and bacteria with the immune system of the patient [98] while the response of the host may vary, ranging from no reaction to excessive reaction, triggering further organ dysfunction.

As explained before, CO is able to regulate and control inflammation, not only in organ dysfunction but also in sepsis [82, 99–101]. This may be of special interest in septic conditions, since phagocytosis of bacteria may re-establish the balance of the immune response. Carbon monoxide is capable of the induction of macrophages, activating autophagic processes for the uptake of bacteria, such as *Escherichia col i*[83]. While specific trials regarding the CO administration in septic humans are still missing, there is a wide amount of data, analyzing the effects of CO in experimental sepsis. These experiments include almost every organ system and ranges from LPS-induced sepsis to myocardial sepsis and ARDS. The experimental data regarding potential treatment options with CO or CO-RMs in septic models include the classical targets molecules of CO, such as the NLRP3 inflammasome or the MAPK [100, 102]. Apart from ARDS, no other septic or septic-like condition has been treated in humans using CO. Although, the origin of sepsis is multidimensional and differs considerably between humans, CO may possess the characteristics to maintain the balance between inflammation and resolution in different organ systems [103].

## Extracorporeal circulation units

Cardiac arrest requiring basic and advanced cardiac life support occurs in approximately 250,000 patients in Europe annually and remains the leading cause of death despite advances in treatment options [104]. Mostly of cardiogenic origin (acute myocardial infarction) [105], survival rates are as low as ~ 10% [106] and if survived, patients suffer from massive constrains in daily life and activity. These include cerebral damage, cognitive deficits, myocardial injury, and limited hemodynamics which determine the patients’ prognosis even if return-of-spontaneous circulation has been achieved. While conventional cardiopulmonary resuscitation strategies did not improve overall survival significantly over the last years, alternative therapies such as extracorporeal CPR (E-CPR) are emerging [107, 108] showing first data of improved survival rates and enhanced neurological outcome after in-hospital-cardiac arrest. Beside the opportunity and promising beneficial effects in using any form of extracorporeal circulation unit, one must be aware of the side effects (e.g., renal failure etc.) [109].

Recently, Wollborn and colleagues introduced a new method on administering carbon monoxide via CO-RM (Beck-1) safely to an extracorporeal circulation unit while separating any transition metal component from the bloodstream of the circulation unit [110]. As a consequence, they were able to demonstrated improved hemodynamics and enhanced cardiac function in a porcine model of cardiac arrest and subsequent E-CPR with CO supplementation [111]. Macro- and microcirculation showed CO-dependent constancy against the otherwise harmful ECLS System. Cardiac tissue swelling was reduced as well as the expression of Troponin-T. Furthermore, the authors were able to show that the left ventricular ejection fraction and the VTI of the left ventricle was constantly higher in animals with CO administration during and after ECLS using transesophageal echocardiography. These effects were in line with following results, showing improved renal function after ECLS implementation and CO supplementation. Serum parameters of renal function (creatinine and NGAL) as well as a renal injury score resulting from histological analysis revealed a significant decrease due to CO administration [112]. Although the study was not performed in humans [113], the authors describe a well-established model of a relevant clinical condition. Apart from the usual focus on the molecular mechanism, the authors focused on danger-associated molecular patterns (DAMPS) as the targets for CO. Among these, IL-6, HMGB-1, Cytochrom-C, and HO-1 were analyzed and displayed significant changes due to CO administration. Taken together, a new therapeutic approach of high clinical relevance in treating cardiac arrest and its sequelae has been reported. Regarding the safe and fast onset of CO delivery during extracorporeal resuscitation, a foundation has been created for a new generation of CO delivery protocols with high translational significance.

## Clinical trials initiated

Apart from the many studies performed in humans, research is still ingoing. To-date, there are at least two listed trials, aiming to support the data generated so far. The first study, which is already recruiting patients, again aims to treat the acute respiratory distress syndrome. Two different treatment arms have been initiated in this phase-II trial: the administration of carbon monoxide will be 100 ppm and 200 ppm respectively (NCT02425579 [100 ppm] and NCT03799874 [200 ppm]). Moreover, the first-in-man study to evaluate the effects of oral administration of a formulation to induce HO-1 in order to increase endogenous carbon monoxide production (http://www.hillhurstbio.com/hbi-002). This study will be performed in healthy volunteers and is yet to start recruitment (NCT03926819: A study to assess the safety and pharmacokinetics of HBI-002, an oral carbon monoxide therapeutic in healthy volunteers). It will be most exciting for both trials to discuss the results, which are expected to be present in the next years.

## Summary and conclusions

Carbon monoxide should no longer be considered as a toxic agent only. While being generated endogenously, its effects and its specific molecular mechanism has been investigated in a broad variety of different models and disease conditions. It has been demonstrated that it may be implicated in a safe yet effective manner. Although clinical studies are rare and patient numbers included in these studies are low, carbon monoxide remains one of the most promising substances with great potential regarding the future development of certain critical illnesses.

The timing of CO application may be of crucial interest. A prophylactic treatment (e.g., in organ donor settings like living donation) may alter other target molecules than a therapeutic management of sepsis or AKI. In most cases, a prophylactic treatment is not possible—this is one reason why pre-conditioning experiments vanished over the last years. More likely, patients present with a certain degree of organ dysfunction, thus in need of a therapeutic alternative. In contrast to other experimental agents, CO offers both, the prophylactic conditioning in a known but yet not acute organ dysfunction and the therapeutic treatment option in case of severe organ dysfunction. In most cases of human research, CO has been given as a therapeutic agent, to counteract systemic or organ damage.

Given the large number of published work on CO’s potentially beneficial properties in different organ systems, we should not cold-shoulder CO because of its known toxic effects. Carbon monoxide is an attractive agent while being cheap, simple to administer, and available (in contrast to other gaseous mediators). A wide therapeutic window may open up offering new methods in treating special organ failure either by inhalation or by specific transport of CO-RMs to the tissue of interest.

While carbon monoxide for sure is no magic bullet, it may help to alter especially overwhelming inflammatory conditions in ARDS, AKI, sepsis, transplant settings, or in patient in need of extracorporeal circulation. With the option of different CO-RMs to delivery carbon monoxide tailored to a specific disease to a certain organ or tissue at a particular time, we face the future not only regarding drug development but also in treatment options. While Hooper and colleagues asked “Where is the clinical breakthrough of HO-1 and CO therapeutics?” [114], we should start to give the answers to these and other questions providing larger studies with relevant clinical endpoints.

## Data Availability

Not applicable.
